# CYT997(Lexibulin) induces apoptosis and autophagy through the activation of mutually reinforced ER stress and ROS in osteosarcoma

**DOI:** 10.1186/s13046-019-1047-9

**Published:** 2019-01-31

**Authors:** Zongyi Wang, Fei Yin, Jing Xu, Tao Zhang, Gangyang Wang, Ming Mao, Zhuoying Wang, Wei Sun, Jing Han, Mengkai Yang, Yafei Jiang, Yingqi Hua, Zhengdong Cai

**Affiliations:** Department of Orthopaedics, Shanghai Bone Tumor Institute, Shanghai General Hospital, Shanghai Jiao Tong University School of Medicine, Poster address: 100 Haining Road Shanghai, Shanghai, 20160 China

**Keywords:** Osteosarcoma, CYT997, ER stress, ROS, ERO1A

## Abstract

**Background:**

Osteosarcoma (OS) is a common malignant cancer in children and adolescents and has a cure rate that has not improved in the last two decades. CYT997 (lexibulin) is a novel potent microtubule-targeting agent with various anticancer activities, such as proliferation inhibition, vascular disruption, and cell cycle arrest and apoptosis induction, in multiple cancers. However, the direct cytotoxic mechanisms of CYT997 have not yet been fully characterized.

**Methods:**

We evaluated apoptosis and autophagy in human osteosarcomas after treatment with CYT997 and investigated the underlying mechanisms. To explore relationships, we used the reactive oxygen species (ROS) scavenger N-acetyl cysteine (NAC), PERK inhibitor GSK2606414, ERO1 inhibitor EN460 and mitochondrial targeted protection peptide elamipretide. BALB/c-nu mice were inoculated with 143B tumor cells to investigate the in vivo effect of CYT997.

**Results:**

We explored the efficacy and mechanism of CYT997 in osteosarcoma (OS) in vitro and in vivo and demonstrated that CYT997 potently suppresses cell viability and induces apoptosis and autophagy. CYT997 triggered production of ROS and exerted lethal effects via endoplasmic reticulum (ER) stress in OS cells. NAC attenuated these effects. The PERK inhibitor GSK2606414, which can block the ER stress pathway, reduced ROS production and enhanced cell viability. Moreover, activation of ERO1 in the ER stress pathway was responsible for inducing ROS production. ROS produced by the mitochondrial pathway also aggravate ER stress. Protection of mitochondria can reduce apoptosis and autophagy. Finally, CYT997 prominently reduced tumor growth in vivo.

**Conclusions:**

This study suggests that CYT997 induces apoptosis and autophagy in OS cells by triggering mutually enhanced ER stress and ROS and may thus be a promising agent against OS.

**Electronic supplementary material:**

The online version of this article (10.1186/s13046-019-1047-9) contains supplementary material, which is available to authorized users.

## Background

Osteosarcoma (OS), known as sarcoma derived from bone-forming mesenchymal cells, is most commonly diagnosed among children and adolescents between 15 and 19 years of age [1]. In the past three decades, new technologies have facilitated the early diagnosis of OS, and improvements in surgery and chemotherapy have increased the 5-year survival rate from less than 20% to 65–75%. However, as the cure rate has remained stagnant over the past 20 years [2], we urgently need new methods to treat this disease.

Microtubules are an integral part of the cell cytoskeleton, participating in the maintenance of cell structure and providing a platform for intracellular transport and a variety of cellular processes. CYT997 is a potent orally bioavailable microtubule-disrupting agent that also inhibits tubulin polymerization. CYT997 has been used in phase I clinical trials of non-small cell lung cancer (NSCLC), colorectal cancer, pancreatic adenocarcinoma and breast cancer [3–5]. However, there are only a few studies on the effects and intrinsic molecular mechanisms of CYT997 in OS.

The endoplasmic reticulum (ER) consists of vesicles and membranous tubules and is connected to the plasma membrane and also communicates with the outer nuclear membrane. The ER has many essential functions including the synthesis, modification and processing of proteins [6, 7]. Live cell imaging studies have revealed that the ER and microtubule connections are critical for ER remodeling and distribution and that the ER and microtubules participate in extensive functional communication [8]. Conditions of cellular stress can lead to the accumulation of unfolded proteins in the ER and trigger the unfolded protein response (UPR), a double edge sword that can protect cells or, if the stress is too severe to be overcome, trigger apoptosis or autophagy [9, 10].

Reactive oxygen species (ROS) comprise a series of byproducts produced by aerobic cells during metabolism that have important roles in biochemical functions. Normally, ROS produced by mitochondrial redox chain can maintain a balance between production and removal. However, an excess of ROS yields toxic effects on DNA and proteins and triggers apoptosis [11]. Endoplasmic reticulum oxidoreductin 1 (ERO1) is a eukaryotic flavin adenine nucleotide-containing enzyme located in the ER that transfers electrons from reduced protein disulfide isomerase (PDI) to the terminal acceptor. ERO1 is known as a producer of H2O2, which can increase the ROS burden in cells. ERO1 expression is increased when ER stress occurs [12]. Additionally, many studies have reported that increases in ROS lead to misfolded proteins and ER stress [13]. We hypothesize that ER stress and ROS are mutually connected and reinforce each other’s production.

In the present study, we used both in vitro and in vivo models to investigate whether CYT997 can stimulate UPR and upregulate ERO1 to produce ROS, and excessive ROS production by mitochondrial and ER exacerbates UPR, eventually leading to cell death. CYT997 may be a promising chemotherapeutic drug for OS patients.

## Methods

### Cells and cell culture

The human OS cell lines 143B, SJSA, MG63, and U2OS were purchased from American Type Culture Collection (ATCC; Manassas, VA, USA). All cell lines were cultured in Dulbecco’s modified Eagle’s medium (HyClone, Logan, UT, USA) with 10% fetal bovine serum (FBS; Thermo, Waltham, MA, USA) plus 1% penicillin/streptomycin (Thermo, Waltham, MA, USA). The cells were maintained at 37 °C in a humidified incubator with 95% air and 5% CO2.

### Reagents and antibodies

CYT997 (MF: C_24_H_30_N_6_O_2_, MW: 434.53, purity: 99.46%) was purchased from Selleckchem (Houston, TX, USA) and dissolved in dimethyl sulfoxide (DMSO) to prepare a 40 mM stock solution, which was stored at − 80 °C. DMSO was obtained from Sigma-Aldrich (St. Louis, MO, USA). 2′,7′-Dichlorodihydrofluorescein diacetate (DCFH-DA), N-acetylcysteine (NAC), 3-methyladenine (3-MA), chloroquine (CQ) and GSK2606414 were purchased from Sigma-Aldrich. EN460 was purchased from MedchemExpress (Monmouth Junction, NJ, USA). Antibodies against PARP, C-PARP, CASPASE-4, LC3B-I/II, BECLIN-1, PERK, P-PERK, EIF2A, P-EIF2A, CHOP, ERO1-Lα, and GAPDH were purchased from Cell Signaling Technology (Beverly, MA, USA).

### Cell viability assay

Cells (5 × 10^4^/ml) were seeded in 96-well plates overnight and then treated with various concentrations of CYT997 (0, 20, 40, 80, 160, or 320 nM) (the concentration of DMSO was kept at < 0.1%). After 24 or 48 h, 90 μl fresh medium without FBS and with 10 μl of Cell Counting Kit-8 (CCK-8) solution (Dojindo, Tokyo, Japan) was added to each well for 1.5 h at 37 °C. Absorbance was measured at 450 nm using a microplate reader (iMark; Molecular Devices, Sunnyvale, USA).

### Colony formation assay

Five hundred cells per well were seeded in six-well plates. After 24 h, fresh medium containing CYT997 (0, 40, 80, or 160 nM) was added to each well for 15 days until the cells grew into visible colonies. The cells were washed 3 times with PBS and fixed with 4% paraformaldehyde and stained with crystal violet for 15 min. The colonies that contained more than 50 cells were counted.

### Apoptosis analysis by flow cytometry

Cells (5 × 10^5^/ml) were seeded in six-well plates for 24 h and treated with CYT997 (0, 40, 80, or 160 nM) for 24 h. The cells were harvested, washed twice with cold phosphate-buffered saline (PBS) and resuspended in 1× binding buffer. The cells were then incubated with Annexin V-FITC/PI (Invitrogen Life Technologies, Carlsbad, CA, USA) for 15 min in the dark at room temperature following the manufacturer’s protocol and analyzed using an Accuri C6 flow cytometer (BD Biosciences, Mountain View, CA, USA).

### Western blotting

Cells and tissues were lysed in ice-cold radioimmunoprecipitation (RIPA) buffer containing a protease and phosphatase inhibitor cocktail (Sigma-Aldrich) for 30 min, and the lysates were centrifuged at 12,000 rpm for 20 min at 4 °C. The supernatant was collected, and protein concentrations were quantified using a BCA protein assay kit (Thermo Scientific, Fremont, CA, USA) according to the manufacturer’s instructions. Equal amounts of protein were separated by SDS-PAGE (10–12%) at 100 V for 1.5 h and transferred to polyvinylidene difluoride (PVDF) membranes (Millipore, Billerica, MA, USA). The membranes were incubated in 5% nonfat milk for 1 h at room temperature and incubated overnight at 4 °C with specific primary antibodies. The next day, the membranes were washed 3 times with TBST for 10 min and then incubated with secondary antibodies (Sigma-Aldrich) for 1 h at room temperature. After washing three times, signals were detected using an enhanced chemiluminescence kit (Millipore). The WB results are provided in the Additional file 1.

### GFP-LC3 puncta and LysoTracker red staining assay

OS cells were cultured in six-well plates and transfected with 2 μg/ml GFP-LC3-encoding plasmids using Lipofectamine 2000 (Invitrogen, Carlsbad, CA, USA) following the manufacturer’s protocol. After 24 h of transfection, the cells were treated with or without 80 nM CYT997 for 24 h. The cells were washed twice with PBS, incubated with 50 nM LysoTracker Red DND-99 (Invitrogen) in the dark for 30 min at 37 °C, washed twice with PBS, fixed with 4% paraformaldehyde for 20 min and incubated with DAPI for 5 min. Images were obtained using a confocal laser scanning microscope (Leica, Germany) with LAS V4.3 software.

### Intracellular ROS and superoxide detection

ROS and superoxide production were detected using the peroxide-sensitive fluorescent probe DCFH-DA and MitoSOX Red dye (Invitrogen). Cells were seeded in six-well plates at a density of 5 × 10^5^/ml and exposed to CYT997 (80 nM) for 12 h. The cells were incubated with 10 μM DCFH-DA or 1 μM MitoSOX Red dye in fresh medium for 20 min and washed three times with PBS. ROS and superoxide levels were determined by fluorescence microscopy and confocal laser scanning microscopy (Leica, Wentzler, Germany), and the intensity of DCFH-DA was detected by flow cytometry (BD Biosciences, San Jose, CA, USA).

### Transmission electron microscopy

Cells were treated with CYT997 (80 nM) for 24 h and fixed with 2.5% glutaraldehyde solution (Sigma-Aldrich, St. Louis, MO, USA, G5882) at 4 °C overnight and then fixed in 1% buffered osmium tetroxide for 1.5 h. The dehydrated cells were embedded and stained with uranyl acetate. Representative areas were chosen for ultrathin sectioning detected by transmission electron microscopy (TEM) (FEI Tecnai G2 12, Eindhoven, The Netherlands).

### Plasmid transfection

Transfection was performed using Lipofectamine 3000 Transfection Reagent (Invitrogen, 11,668–019) following the manufacturer’s protocol. To generate an ERO1 expression plasmid, human ERO1-Lα was cloned into the pcDNA3.1 vector by Cyagen Biosciences Inc. (Shanghai, China). Short hairpin RNAs (shRNAs) targeting ATG5 and ATG7 were purchased from Genomeditech Inc. (Shanghai, China). 143B cells were cultured in six-well plates and transfected with plasmids for 24 h. After designated treatments, live cell images were obtained using a fluorescence microscope (Leica, Wentzler, Germany). Protein overexpression efficiency was assessed by immunoblotting.

### Hydrogen peroxide assay

The concentration of intracellular H2O2 was measured using a hydrogen peroxide assay kit (Bio-Rad, CA, USA) according to the manufacturer’s instructions. A total of 1 × 10^5^ cells were lysed in 100 μl of lysis buffer, and they were collected by centrifugation at 12,000×g for 5 min. Supernatants (50 μL) were mixed with reaction reagent (100 μl) for 30 min at room temperature and measured at a wavelength of 560 nm. The concentration of H2O2 was calculated by a standard curve generated with H2O2 standard solutions.

### In vivo mouse study

Four-week-old female BALB/c-nu mice (Shanghai Slac Laboratory Animal Co., Ltd., Shanghai, China) were purchased and housed in a standard animal laboratory with free access to water and food. 143B cells were harvested and washed three times with cold PBS. Then, 100 μl cell suspension at a 1 × 10^7^/ml density was injected into the medullary cavity of the tibia. When the tumors in the tibia were macroscopically visible, the mice were randomly divided into four groups: control group, 3-MA and CYT997 single treated group and 3-MA plus CYT997 treated group (five mice in each group). The mice were sacrificed after 28 days, and the tumors were dissected, weighed, stored in liquid nitrogen or fixed in 4% paraformaldehyde 24 h for further analysis. The weight of tumor is recorded as the weight of the tumor-bearing limb minus the weight of the other side. All animal procedures were performed following protocols approved by the Animal Care and Use Committee of Shanghai General Hospital and Shanghai Jiaotong University.

### Hematoxylin and eosin staining and immunohistochemistry

Primary tumors, hearts, livers, lungs and kidneys were fixed overnight, embedded in paraffin and then cut into serial sections (4-μm thick). The sections were subjected to hematoxylin and eosin (H&E) staining. Representative images were acquired using a microscope (Leica). For immunohistochemical staining, slides were deparaffinized in xylene and rehydrated with graded alcohol and incubated in 3% H2O2 to block endogenous peroxidase activity. The slides were boiled for 30 min in 10 mM sodium citrate (pH 6.0) for antigen retrieval and then blocked in 5% BSA for 30 min, followed by incubation with antibodies against p-eif2α and ERO1-Lα at the appropriate dilution at 4 °C overnight in a humidity chamber. Afterward, the slides were washed three times with PBS and then incubated with secondary antibodies for 30 min at room temperature. Immunoreactivity was visualized using a Vectastain Elite DAB Kit (Vector Laboratories, Burlingame, CA, USA).

### Statistical analysis

Data are presented as the mean ± SD (vertical error bars) from three independent experiments, except for the animal experiments models, and a *P* value less than 0.05 was considered statistically significant. Treatment effects among groups were evaluated using Student’s t test, Fisher’s exact test or one-way ANOVA, and no data points in our study were excluded. Statistical analyses were performed using SPSS version 19.0 software (IBM Corporation, Chicago, USA).

## Results

### CYT997 inhibits viability and induces apoptosis in osteosarcoma cells

**Fig. 1:**
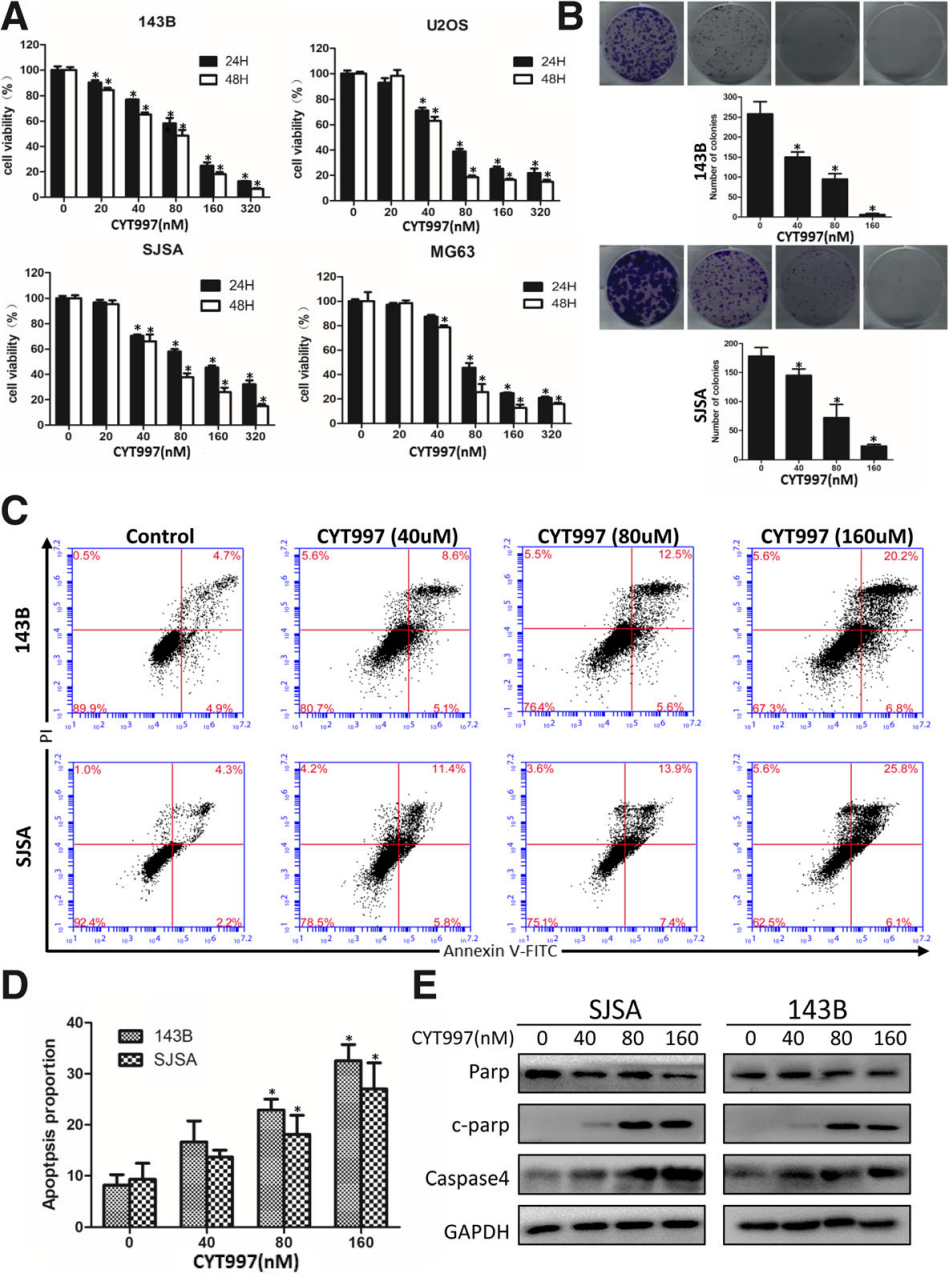
CYT997 inhibited cell proliferation and induced apoptosis in human osteosarcoma cells. **a**. 143B, SJSA, U2OS, and MG63 osteosarcoma cell lines were treated with CYT997 (0, 20, 40, 80, 160 and 320 μM) for 24 and 48 h. Cell viability was measured by CCK-8 assays. **b**. 143B and SJSA cells treated with CYT997 (0, 40, 80, 160 μM). Colony formation was evaluated by colony formation assays. **c**-**d** 143B and SJSA cells were treated with CYT997 for 24 h and analyzed using PI/Annexin V-FITC flow cytometry. Histograms indicate the proportion of apoptotic cells from three separate experiments. **e** Cells were treated with various concentrations of CYT997 for 24 h, and apoptosis-related proteins such as cleaved PARP, and caspase-4 were analyzed by western blotting. *P < 0.05, significantly different compared with the control group

First, to determine whether CYT997 affects OS cell proliferation, we treated OS cell lines 143B, SJSA, U2OS and MG63 with various concentrations of CYT997 for 24 and 48 h, and cell viability was measured using the CCK-8 assay (Fig. 1a). The OS cell survival rate displayed a significant dose- and time-dependent decrease. The IC50 values of 143B, SJSA, U2OS and MG63 cells were 92.52, 103.21, 65.87 and 71.93 nM, respectively, at 24 h and 78.71, 73.64, 52.93, and 67.38 nM, respectively, at 48 h. Subsequently, we confirmed colony formation in 143B and SJSA cells as previously described (Fig. 1b). The experimental results were consistent with the CCK-8 assay results, indicating that CYT997 can significantly inhibit OS cell proliferation.

We then determined the apoptosis-inducing abilities of CYT997 in 143B and SJSA cells using flow cytometry analysis with PI/Annexin-FITC staining to examine apoptosis induction by CYT997. As shown in Fig. 1c and d, the proportion of apoptotic cells was significantly increased in a dose-dependent manner after treatment with CYT997.

To further determine which pathway mediates CYT997-induced apoptosis, we investigated the expression of apoptotic-related proteins, including caspase-4 and c-PARP, by western blotting in 143B and SJSA cells (Fig. 1e and Additional file 1: Figure S2 AB). Caspase-4 is a paralog of caspase-12 and is associated with ER stress-induced apoptosis [14]. An obvious increase in expression of c-PARP and caspase-4 was found with different concentrations of CYT997. Our results demonstrated that CYT997 dramatically inhibits OS cell proliferation and induces apoptosis.

### CYT997 induces autophagy to promote cell survival

**Fig. 2:**
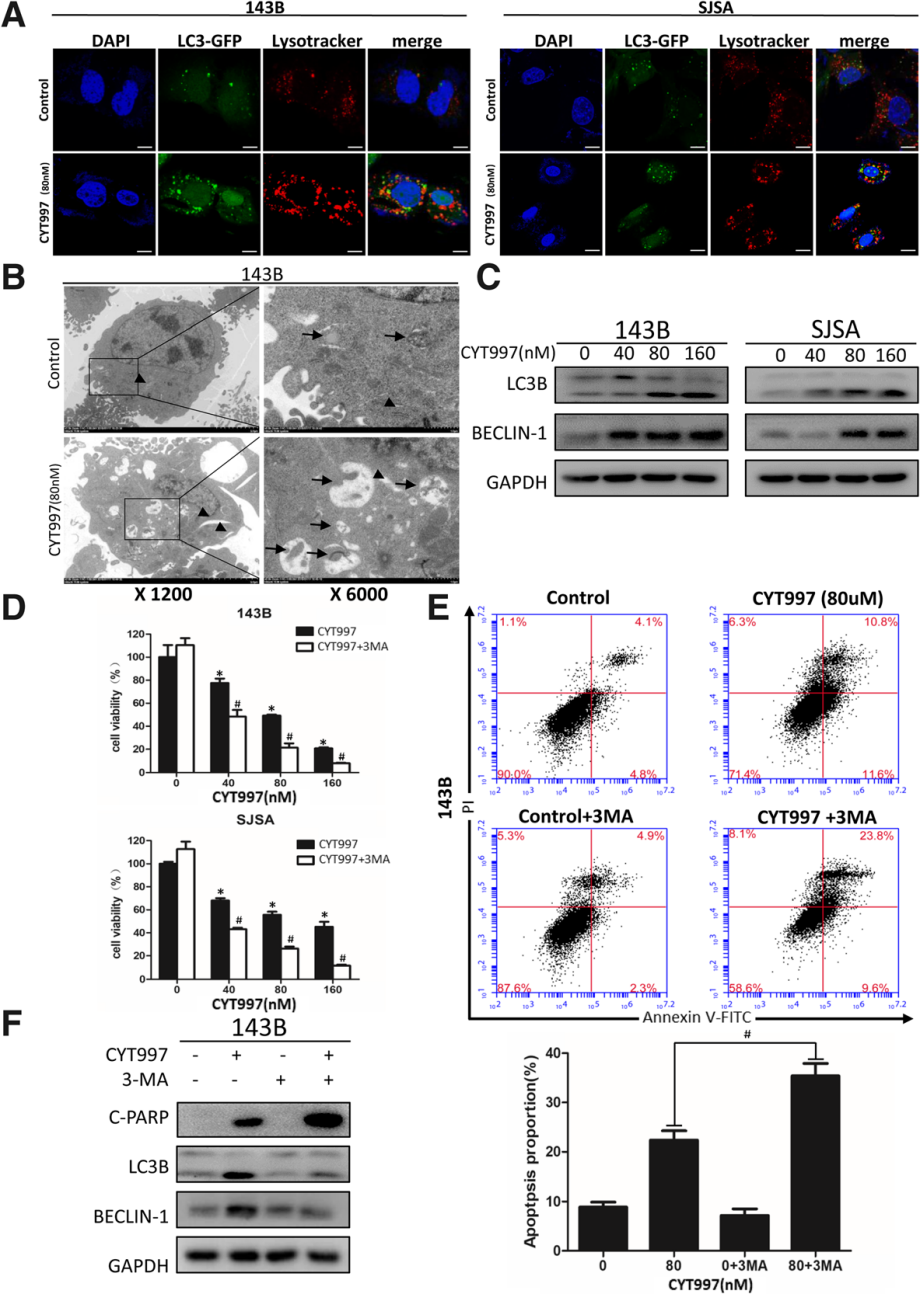
CYT997 induced autophagy in OS cells, and inhibition of autophagy increased CYT997-induced apoptosis. **a** Osteosarcoma cell lines 143B and SJSA were transiently transfected with GFP-LC3-encoding plasmids for 24 h, treated with or without CYT997 (80 nM) for 24 h and stained with LysoTracker Red DND-99 (50 nM). Green color represents the formation of autophagosomes, and red color shows cellular acidic compartments, indicative of lysosomes and autolysosomes. Colocalization of autophagosomes and lysosomes was examined by confocal microscopy. Scale bars = 20 μm. **b** CYT997 induced accumulation of autophagosomes in osteosarcoma cells, as shown in the electron micrographs. Arrows indicate autophagosomes, and arrowheads indicate ER. **c** Osteosarcoma cells were treated with CYT997 (80 nM) for 24 h. Autophagy-related proteins, LC3B and beclin-1, were analyzed by western blotting. **d** 143B and SJSA cells were preincubated with 3-methyladenine (3-MA) (5 mM) for 2 h and then treated with CYT997 (80 nM) for 24 h, followed by cell proliferation detection using CCK-8 assays. **e** Osteosarcoma cells were preincubated with 3-MA (5 mM) and then treated with CYT997(80 nM) for 24 h and analyzed using PI/Annexin V-FITC flow cytometry. Histograms indicate the proportion of apoptotic cells from three separate experiments. **f** Cells were treated with 80 nM CYT997 and 3-MA for 24 h, and the levels of c-PARP, LC3B and Beclin-1 were assessed by western blotting. *P < 0.05, significantly different compared with the control group. # *P* < 0.05, significantly different compared with the CYT997 treatment group

We next determined whether CYT997 can induce autophagy in OS cells. First, 143B and SJSA were transfected with GFP-LC3-encoding plasmids to analyze the formation of autophagosomes [15], and we used LysoTracker Red dye to label cellular acidic vesicular organelles (AVOs) such as lysosomes [16]. Cells treated with CYT997 exhibited more acidic compartments in the cytoplasm and significantly higher numbers of GFP-LC3 puncta than did control cells. Specifically, as shown in Fig. 2a, the merging of green and red fluorescence represents the fusion of lysosomes and autophagosomes; autolysosomes are labeled as yellow puncta, and these yellow puncta were also visibly increased.

**Fig. 3:**
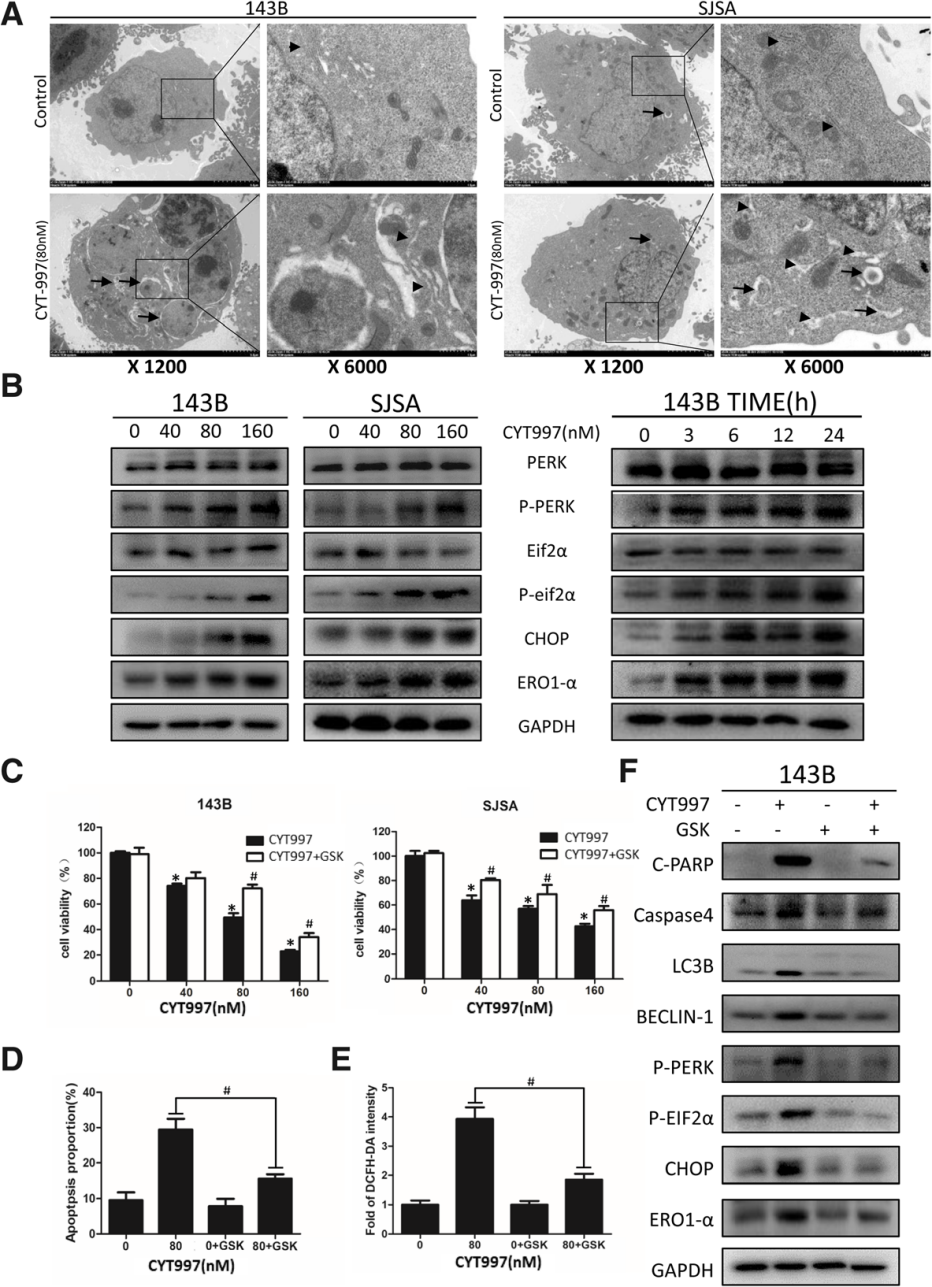
CYT997 induced endoplasmic reticulum (ER) stress in osteosarcoma cell lines, and inhibition of the ER stress pathway decreased CYT997-induced apoptosis, autophagy and reactive oxygen species (ROS) production. **a** Osteosarcoma cell lines 143B and SJSA incubated with CYT997 (80 μM) for 24 h were visualized by electron microscopy; arrowheads point to the ER, and arrows indicate autophagosomes. **b** ER stress-related proteins including PERK, p-PERK, eif2α, p-eif2α, CHOP, and ERO1 were detected by western blotting after incubation of the cells with various concentrations of CYT997 for 24 h or treatment with CYT997 (80 nM) for different durations. **c** 143B and SJSA cells were cotreated with GSK2606414 (2 μM) and different concentrations of CYT997 for 24 h, followed by cell proliferation detection using CCK-8 assays. **d** 143B cells treated with CYT997 (80 nM) and/or GSK2606414 (2 μM) were analyzed using PI/Annexin V-FITC flow cytometry. Histograms indicate the proportion of apoptotic cells from three separate experiments. **e** 143B cells treated with CYT997 (80 nM) and/or GSK2606414 (2 μM) for 24 h, stained with 10 μM 2′,7′-dichlorodihydrofluorescein diacetate (DCFH-DA) at 37 °C in the dark for 30 min. Histograms indicate the fold change in DCFH-DA intensity relative to the control group from three separate experiments. **f** 143B cells were treated with CYT997 (80 nM) and/or GSK2606414 (2 μM) for 24 h, and apoptosis, autophagy and ER stress-related proteins including c-PARP, caspase-4, LC3B, Beclin-1, p-PERK, p-eif2α, CHOP and ERO1 were analyzed by western blotting. *P < 0.05, significantly different compared with the control group. # *P* < 0.05, significantly different compared with the CYT997 treatment group

Second, we used TEM to visualize the ultrastructures of autophagic organelles in OS cells. Compared to those in the control group, large numbers of autophagosomes were observed in the CYT997-treated group (Fig. 2b and Fig. 3a). Furthermore, we assessed expression of autophagy-related proteins including LC3B and Beclin-1 by western blotting and found that CYT997 increased expression of LC3B-II and beclin-1 in a concentration-dependent manner (Fig. 2c and Additional file 1: Figure S2 CD).

Autophagy is a “double-edged sword” in cancer treatment because it can both promote cell survival and cause cell death [17]. To determine whether CYT997-induced autophagy is prosurvival or prodeath, we used 3-MA, CQ and ATG5 and ATG7-targeted shRNA to inhibit autophagy before CYT997 treatment. CCK-8 analysis and PI/Annexin-FITC flow cytometry indicated that pretreatment with autophagy inhibitor enhanced the effect of CYT997 on cell viability and apoptosis (Fig. 2d, e and f and Additional file 2: Figure S1, Additional file 1: S2E). Additionally, we confirmed the results using western blotting. 143B cells pretreated with 3-MA and CQ had reduced levels of LC3B-II and Beclin-1 and increased levels of c-PARP. Similarly, we performed in vivo experiments to study whether inhibition of autophagy could suppress tumor growth. CYT997 plus 3-MA treatment yielded a superior anti-tumor volume effect than either agent alone (Fig. 7). In conclusion, CYT997 induces autophagy in OS cells, and the induced autophagy promotes cell survival.

### CYT997 induces endoplasmic reticulum stress in human osteosarcoma cells

The above data indicate that CYT997 can induce apoptosis and autophagy in human OS cells. However, the underlying mechanisms still need to be elucidated. To this end, we used TEM and found that the ER lumens of 143B and SJSA cells were severely dilated, indicating an increase in ER stress (Fig. 3a). To further clarify this, we used western blotting to measure the expression levels of UPR-related proteins, including p-PERK, p-eif2α and CHOP. In the CYT997-treated group, expression of p-PERK, p-eif2α and CHOP was significantly increased in a concentration- and time-dependent manner (Fig. 3b and Additional file 1: Figure S2 FGH), strongly indicating that CYT997 induces ER stress in human OS cell lines.

To verify the relationship between ER stress and apoptosis and autophagy, we used the PERK inhibitor GSK2606414, which can block the UPR pathway. We cotreated cells with GSK2606414 (2 μM) and CYT997 (80 μM) for 24 h and then performed CCK-8 assays and PI/Annexin-FITC flow cytometry. As shown in Fig. 3c and d, blockage of the UPR pathway significantly attenuated the inhibitory effect of CYT997 on OS cell proliferation and reduced the apoptosis rate. We further employed western blotting for validation, and expression of UPR pathway markers and c-PARP, caspase-4, LC3B-II and Beclin-1 was decreased in the combination treatment group (Fig. 3f and Additional file 1: Figure S2I). Therefore, CYT997 stimulates the ER stress-related PERK/p-eif2α/CHOP axis to induce apoptosis and autophagy in OS cells.

### CYT997 induces reactive oxygen species production in human osteosarcoma cells

**Fig. 4:**
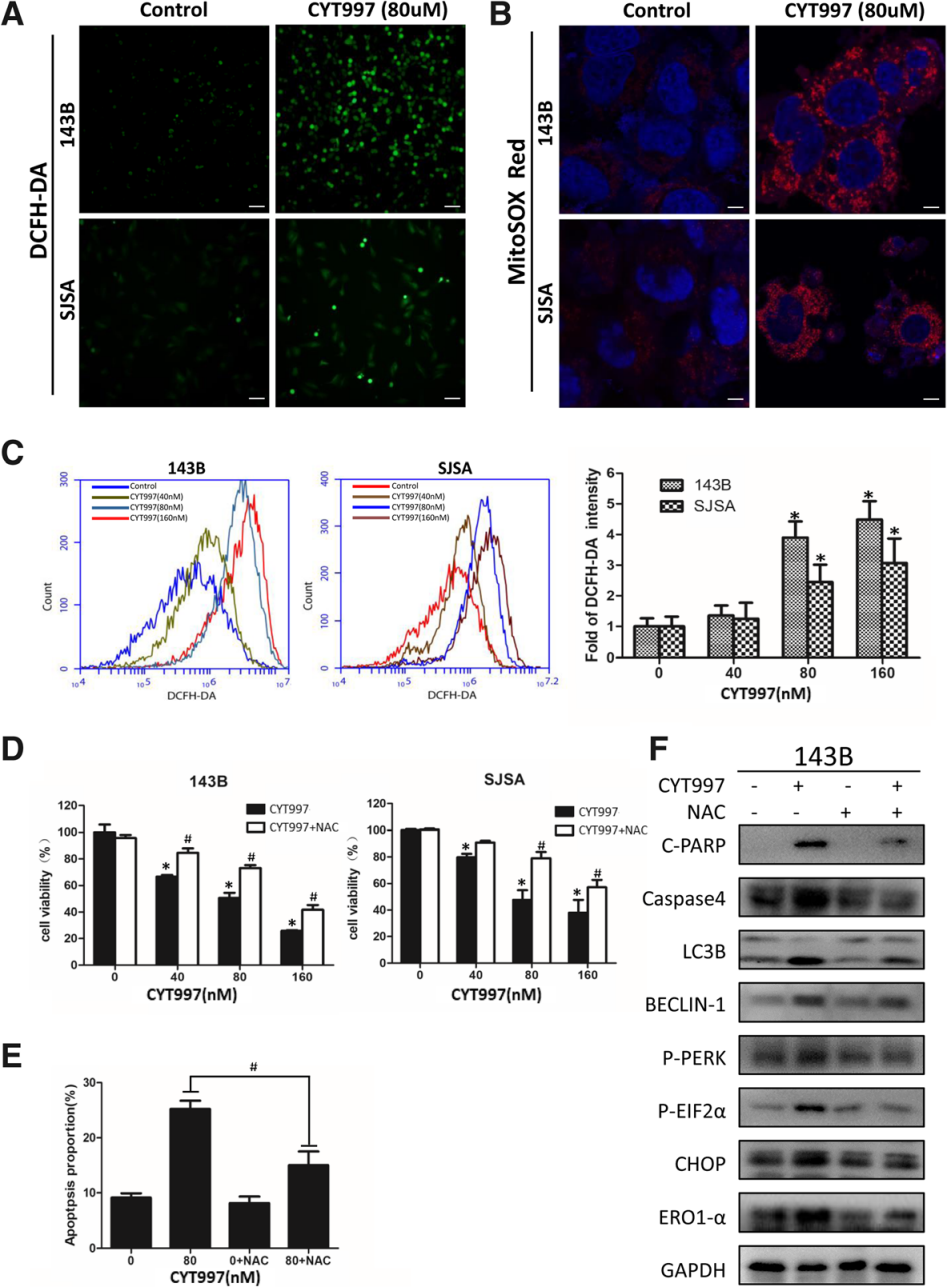
CYT997 induced intracellular reactive oxygen species (ROS) generation, and ROS scavenging decreased CYT997-induced apoptosis, autophagy and endoplasmic reticulum (ER) stress (**a**-**b**). Osteosarcoma cells 143B and SJSA were treated with or without CYT997 (80 nM) for 24 h. Cells were stained with 10 μM 2′,7′-dichlorodihydrofluorescein diacetate (DCFH-DA) or 1 μM MitoSOX Red dye at 37 °C in the dark for 30 min and then observed by fluorescence microscopy and confocal microscopy. Scale bars = 50 and 5 μm. **c** Fluorescence intensity of DCFH-DA was detected using flow cytometry. Histograms indicate the fold change in DCFH-DA intensity relative to the control group from three separate experiments. **d** 143B and SJSA cells were cotreated with NAC (5 mM) and different concentrations of CYT997 for 24 h, followed by the CCK-8 assay for cell proliferation. **e** 143B cells treated with CYT997 (80 nM) and/or NAC (5 mM) analyzed using PI/Annexin V-FITC flow cytometry. Histograms indicate the proportion of apoptotic cells from three separate experiments. **f** 143B cells were treated with CYT997 (80 nM) and/or N-acetyl-L-cysteine (NAC) (5 mM) for 24 h, and apoptosis, autophagy and ER stress-related proteins including c-PARP, caspase-4, LC3B, Beclin-1, p-PERK, p-eif2α, CHOP and ERO1 were analyzed by western blotting. *P < 0.05, significantly different compared with the control group. # *P* < 0.05, significantly different compared with the CYT997 treatment group

ROS have been widely reported to be induced by microtubule-targeting agents [18–20], and they are involved in many cellular functions [21, 22]. As shown in Fig. 4a and b, we used DCFH-DA and MitoSOX Red dye staining after treatment with 80 nM CYT997 for 24 h. Fluorescence microscopy and confocal laser scanning microscopy revealed obvious enhancement in DCFH-DA and MitoSOX Red fluorescent signals relative to those in the control group. To quantitatively measure changes in ROS production, we used flow cytometry to evaluate the intensity of DCFH-DA signals (Fig. 4c). The results indicated that CYT997 induced ROS production in a concentration-dependent manner. To gain further insights into the relationship between ROS and apoptosis, autophagy and ER stress, we cotreated cells with the ROS scavenger NAC (5 mM) and CYT997 (80 μM) for 24 h. Notably, CCK-8 analysis showed that NAC alleviated the cell killing effect, and flow cytometric analysis demonstrated that NAC mitigated CYT997-induced apoptosis and decreased expression of c-PARP and caspase-4 (Fig. 4d, e and f and Additional file 1: Figure S2J). Thus, ROS triggered ER stress, consistent with previous reports [23, 24]. We also found that pretreatment with NAC significantly decreased the levels of UPR-related proteins p-PERK, p-eif2α, CHOP in 143B cells (Fig. 4f). Therefore, CYT997 activates ROS production and induces the UPR pathway, which likely promotes apoptosis and autophagy.

### Reactive oxygen species induction by CYT997 is regulated by the PERK/eif2α/CHOP/ERO1 axis

**Fig. 5:**
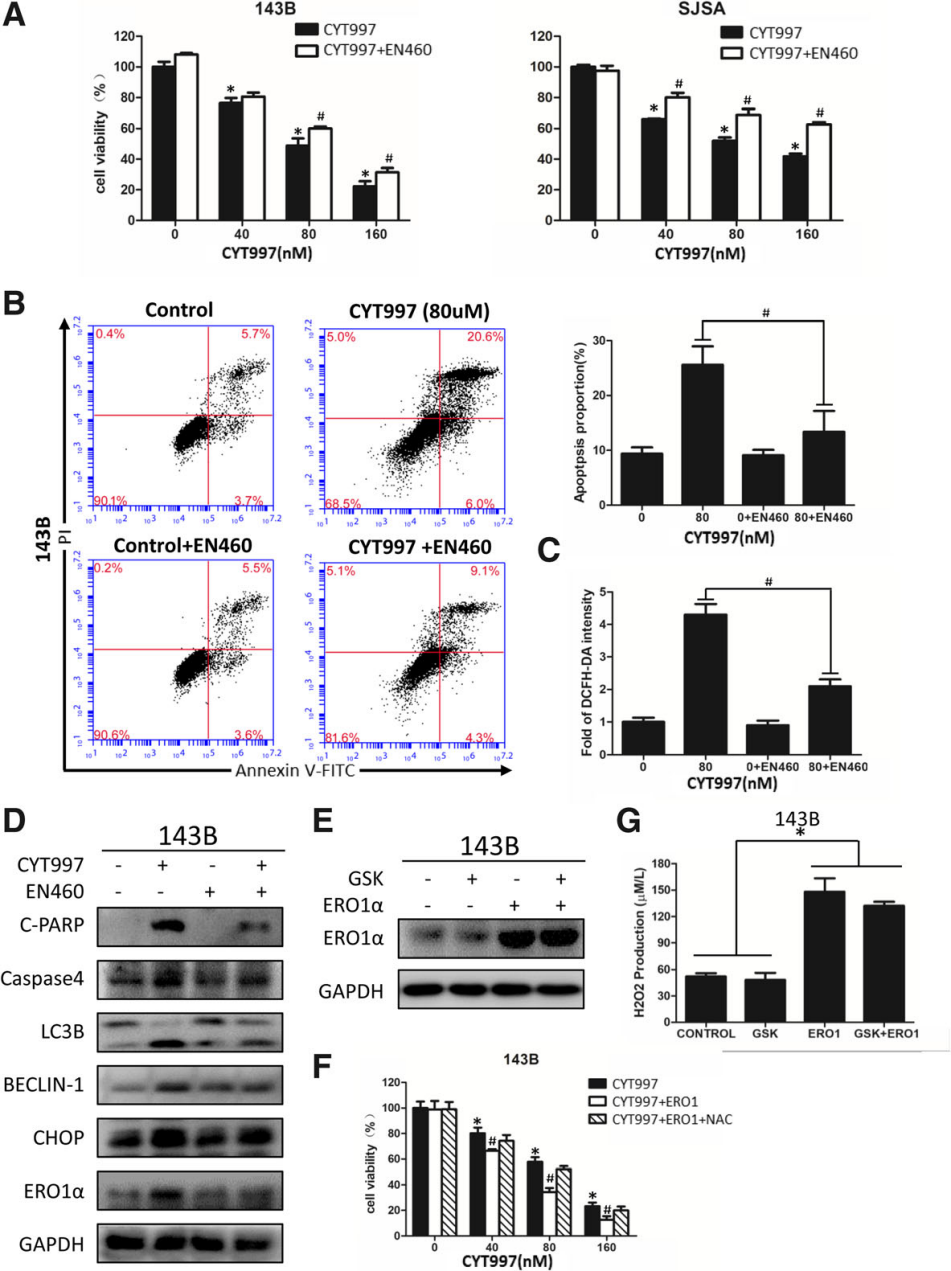
ERO1 regulates reactive oxygen species (ROS) production, and inhibition or overexpression of ERO1 can respectively decrease or increase ROS levels. **a** 143B and SJSA cells were cotreated with EN460 (8 μM) and different concentrations of CYT997 for 24 h, followed by cell proliferation detection using CCK-8 assays. **b** 143B cells treated with CYT997 (80 nM) and/or EN460 (8 μM) 24 h were analyzed using PI/Annexin V-FITC flow cytometry. Histograms indicate the proportion of apoptotic cells from three separate experiments. **c** 143B cells were treated with CYT997 (80 nM) and/or EN460 (8 μM) for 24 h and stained with 10 μM 2′,7′-dichlorodihydrofluorescein diacetate (DCFH-DA) at 37 °C in the dark for 30 min. Histograms indicate the fold change in DCFH-DA intensity relative to the control group from three separate experiments. **d** 143B cells were treated with CYT997 (80 nM) and/or EN460 (8 μM) for 24 h, and apoptosis, autophagy and ER stress-related proteins including c-PARP, caspase-4, LC3B, Beclin-1, CHOP and ERO1 were analyzed by western blotting. **e** 143B cells were transfected with ERO1-encoding vectors and then treated with or without GSK2606414 for 24 h. Protein levels of ERO1 were detected by western blotting. **f** 143B cells transfected with ERO1-encoding vectors were treated with different concentrations of CYT997 and 5 nM of NAC for 24 h, followed by cell proliferation detection using CCK-8 assays. **g** 143B cells were transfected with ERO1-encoding vectors and treated with or without GSK2606414 (2 μM) for 24 h. H2O2 levels were then measured using a hydrogen peroxide assay kit. Histograms indicate the fold change in H2O2 concentration relative to the control group from three separate experiments. *P < 0.05, significantly different compared with the control group. # *P* < 0.05, significantly different compared with the CYT997 treatment group

Next, we investigated the role of ER stress activation in CYT997-induced ROS production. ERO1 is a producer of H2O2 in the UPR pathway and is regulated by CHOP [12, 25]. We pretreated OS cells with 8 μM EN460, an inhibitor that selectively interacts with the active form of ERO1 [26], followed by CYT997 exposure for the next 24 h. CCK-8 and flow cytometric assays showed that EN460 effectively attenuated apoptosis induced by CYT997 (Fig. 5a and b). We measured the DCFH-DA intensity after the combination treatment of cells with CYT997 (80 nM) and EN460 (8 μM) or GSK2606414 (2 μM) for 24 h. Flow cytometry showed that inhibition of ERO1 or blockade of the ER stress pathway decreased the ROS production induced by CYT997 (Figs. 5c and 3e). In addition, EN460 observably decreased expression of apoptosis- and autophagy-related proteins but had no significant effect on CHOP expression (Fig. 5d and Additional file 1: Figure S2K). To further confirm whether ERO1 is closely associated with CYT997-induced ROS production and cell death, we transfected 143B cells with ERO1-encoding plasmids, and we confirmed by western blotting that ERO1 expression was elevated even when the UPR pathway was blocked (Fig. 5e). Based on CCK-8 assay results, ERO1 overexpression also markedly enhanced the killing effect of CYT997 on 143B cells. This killing effect was modulated by NAC (Fig. 5f), and H2O2 levels dramatically increased even after the ER stress was blocked by GSK2606414 (Fig. 5g). Therefore, ERO1 regulates H2O2 production, whereas inhibition or overexpression of ERO1 respectively decreases or increases ROS-induced oxidative stress.

### ROS produced by the mitochondrial pathway also aggravate ER stress

**Fig. 6:**
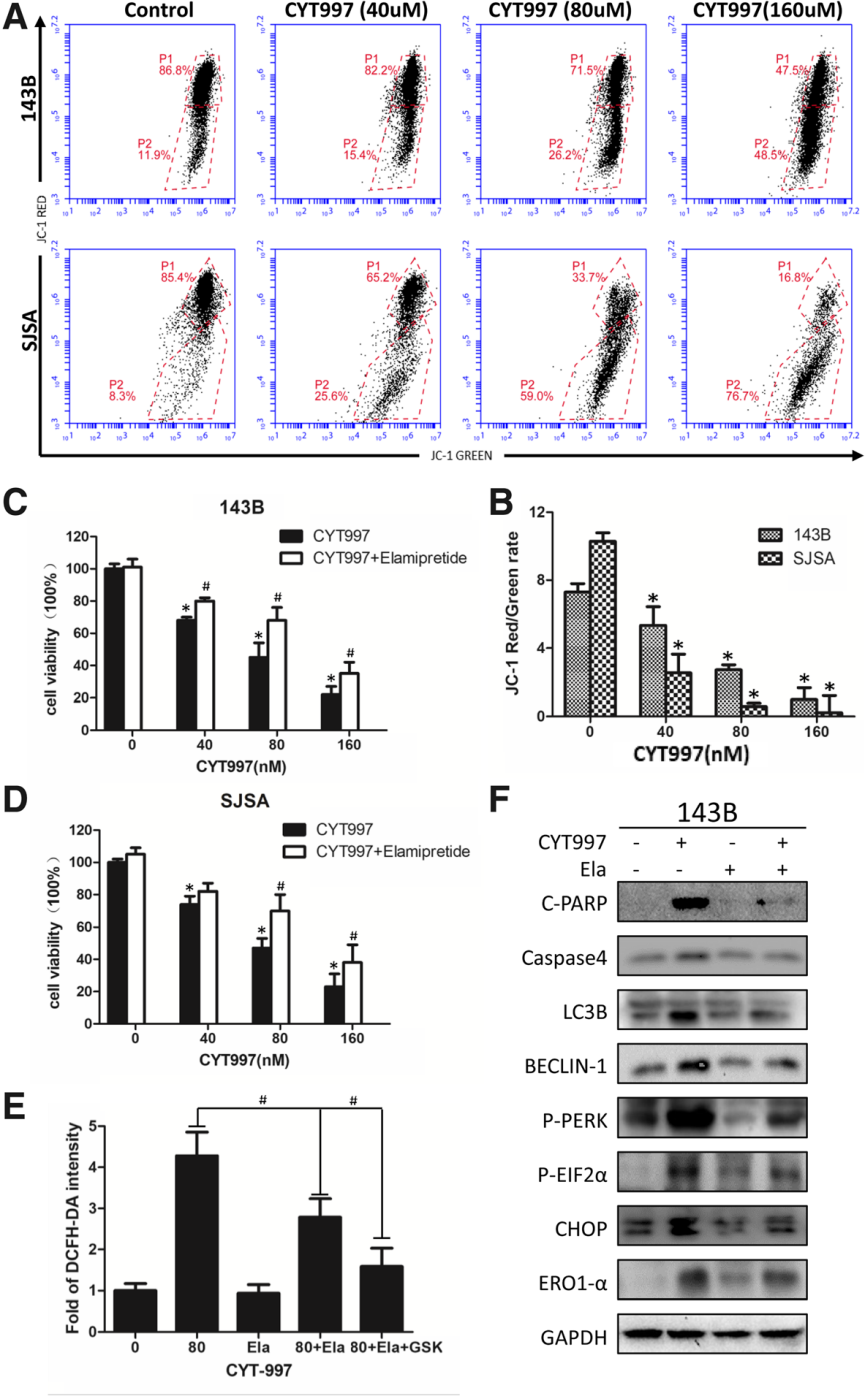
ROS produced by the mitochondrial pathway also aggravate ER stress. **a**, **b**) JC-1 staining by flow cytometry was used to measure the mitochondrial membrane potential (MMP) after CYT997 treatment. Histograms indicate the red/green ratio of JC-1 fluorescence. **c**, **d** 143B and SJSA cells were cotreated with Elamipretide (10 nM) and different concentrations of CYT997 for 24 h, followed by cell proliferation detection using CCK-8 assays. **e** 143B cells were treated with CYT997 (80 nM), Elamipretide(10 nM) and/or GSK2606414 (2 μM) for 24 h and stained with 10 μM 2′,7′-dichlorodihydrofluorescein diacetate (DCFH-DA) at 37 °C in the dark for 30 min. Histograms indicate the fold change in DCFH-DA intensity relative to the control group from three separate experiments. **f** 143B cells were treated with CYT997 (80 nM) and/or Elamipretide (10 nM) for 24 h, and apoptosis, autophagy and ER stress-related proteins including c-PARP, caspase-4, LC3B, Beclin-1, p-PERK, p-eif2α, CHOP and ERO1 were analyzed by western blotting. *P < 0.05, significantly different compared with the control group. # *P* < 0.05, significantly different compared with the CYT997 treatment group

Inhibition of ER stress does not completely remove ROS (Fig. 3e), we continue to explore the role of mitochondria in the treatment of CYT997. To discover the source of ROS, we measured the mitochondrial membrane potential (MMP) with the sensitive fluorescent Probe JC-1 and observed a sharp decrease in the red/green fluorescence ratio (Fig. 6a and b) which indicates that the mitochondria of the cells have undergone severe damage after CYT997 treatment. Then we used elamipretide to protect the mitochondria and reduce the ROS produced by redox chain. CCK-8 showed that elamipretide effectively attenuated apoptosis induced by CYT997(Fig. 6c and d). In addition, elamipretide decreased expression of apoptosis-, autophagy- and ER-stress- related proteins (Fig. 6f and Additional file 1: Figure S2L). We used flow cytometry measure the DCFH-DA intensity after the combination treatment of cells with CYT997 (80 nM) and elamipretide (10 nM) or GSK2606414 (2 μM) for 24 h. showed that protecting mitochondria can reduce ROS production, and blocking ER stress at the same time significantly reduces DHCF-DA levels (Fig. 6e).

### CYT997 inhibits the growth of osteosarcoma in vivo

**Fig. 7:**
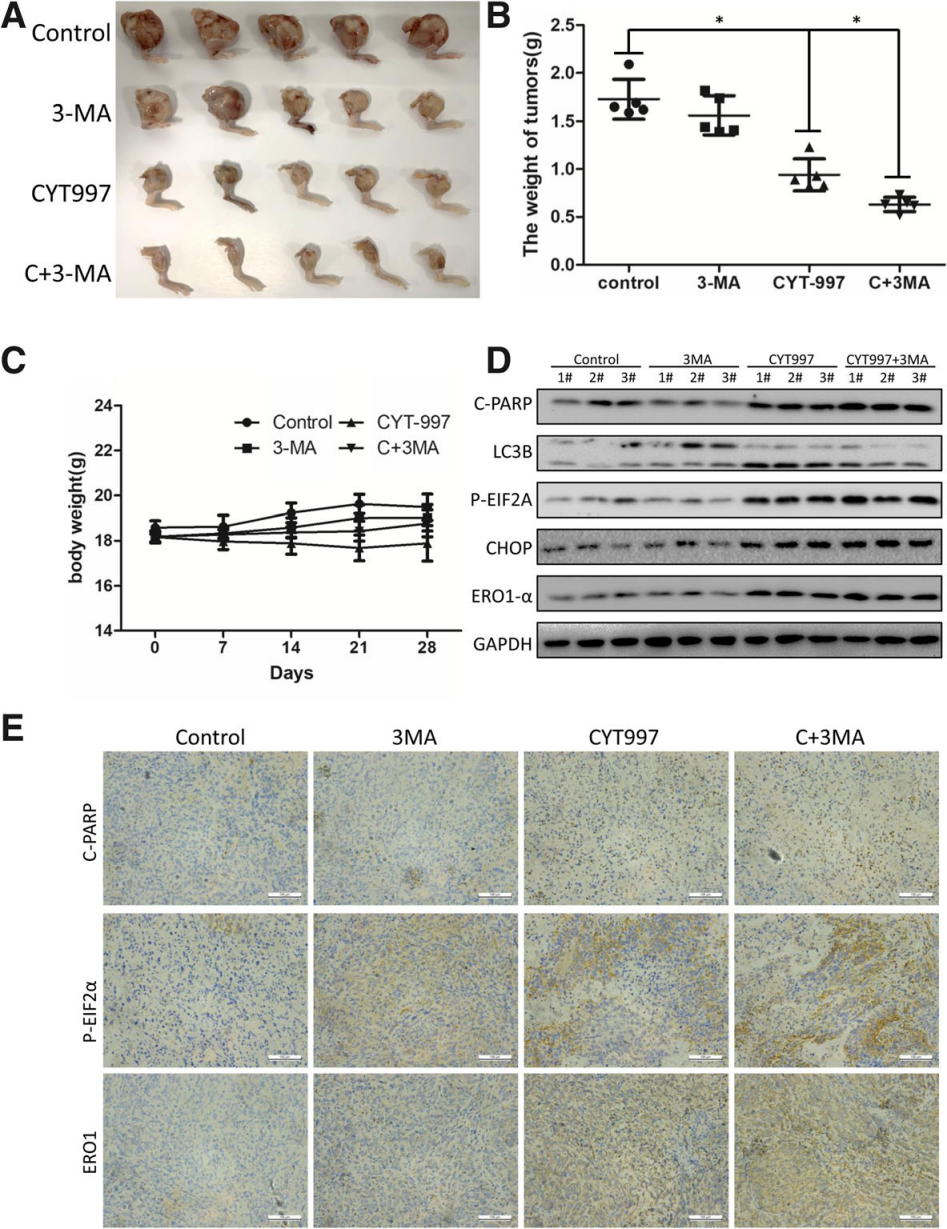
CYT997 suppressed the growth of human osteosarcoma xenografts in vivo, and 3-MA could enhance the in vivo anti-tumor effect. **a** BALB/c-nu mice bearing 143B human orthotopic osteosarcoma xenografts were divided into four groups: [1] mice treated with PBS only, [2] mice treated with 3-MA only, [3] mice treated with CYT997 only and [4] mice treated with CYT997 plus 3-MA. CYT997 plus 3-MA showed more significant anti-tumor effects than did CYT997-only treatment. **b** Tumor weights of the tumors in the four groups were recorded. **c** The weight of mice in the two groups were recorded. **d** Levels of c-PARP, LC3B, p-eif2α, CHOP, and ERO1 in tumor xenograft tissues were measured by western blotting. **e** Immunohistochemical staining of tumor specimens. Scale bars = 50 μm *P < 0.05, significantly different compared with the control group

**Fig. 8:**
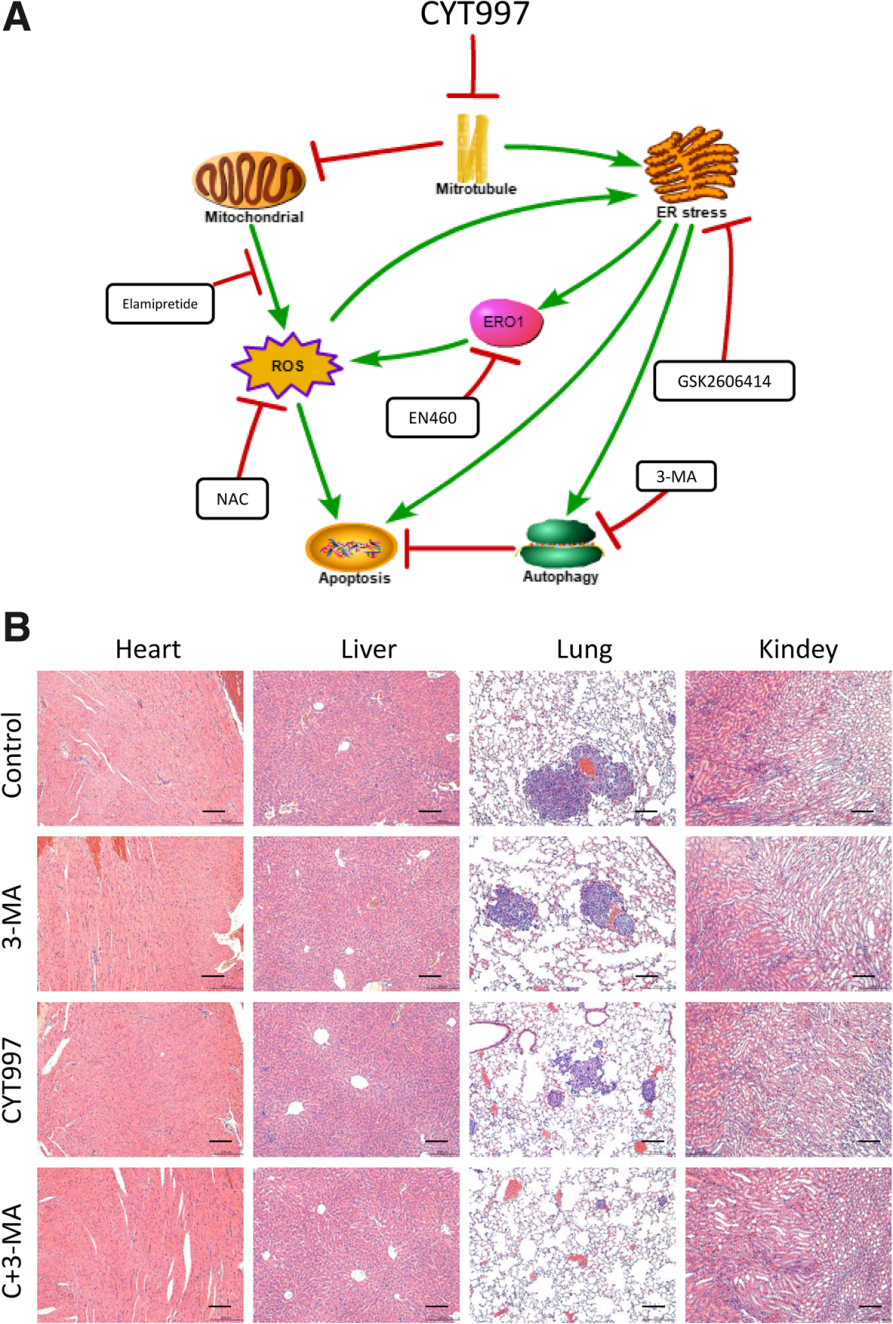
**a** Proposed mechanism of lexibulin-induced apoptosis and autophagy in osteosarcoma cells. **b** H&E staining of important organs. Scale bars = 50 μm

To determine the therapeutic effect of CYT997 in vivo, we used a 143B orthotopic xenograft model in nude mice. When the mean tumor volume reached 80 mm^3^, the nude mice were randomly assigned to four groups: control group, 3-MA(5 mg/kg) and CYT997 (15 mg/kg) single treated group and 3-MA plus CYT997 treated group. The drugs were intraperitoneally administered every day for 28 days. As shown in Fig. 7a and b, compared with the control, CYT997 administration resulted in significantly inhibited tumor growth, Moreover, CYT997 plus 3-MA treatment contributed a superior anti-tumor effect. However, there was no notable difference in body weight between the two groups (Fig. 7c). Western blotting showed that CYT997 and 3-MA treatment increased expression of apoptosis-, autophagy- and ER stress-related proteins (Fig. 7d and Additional file 1: Figure S2M). Immunohistochemical staining demonstrated an increase in necrotic areas and positive staining for C-PARP, Caspase-4, p-eif2α and ERO1 in CYT997-treated tumor tissues (Fig. 7e). H&E staining showed no major organ-related toxicities in the CYT997 and/or 3-MA treated group (Fig. 8b). Thus, CYT997 safely exerts potent antitumor effects of OS in vivo.

## Discussion

As microtubules play an important role in cell growth, motility, division, intracellular trafficking, and cytoskeletal formation, targeting of microtubules has long been used as an effective strategy for cancer treatment. A series of clinical chemotherapeutic drugs, such as vinblastine, docetaxel and paclitaxel, can affect microtubule aggregation or depolymerization in cells. However, these drugs have obvious limitations, such as a lack of good biostability and serious side effects [27–29]. CYT997, an orally stable microtubule polymerization inhibitor discovered by Christopher J. Burns, has potent antitumor activities against various cancer cell lines in vitro and in vivo [4]. Mechanisms of CYT997 include induction of cell cycle arrest, inhibition of angiogenesis, induction of vascular shutdown, perturbation of the cellular architecture, and induction of apoptosis and autophagy in many cancer cell lines [3, 30–32]. We also found that CYT997 can cause cell cycle arrest and increase expression of CyclinB1, p-cdc2 and P21, which are relevant to G2/M phase arrest [33, 34] (data not shown). Hence, in the present study, we demonstrated the therapeutic efficacy of CYT997 in OS and further revealed the possible underlying mechanisms. Indeed, CYT997 suppressed OS cell proliferation and induced ER stress and ROS production, both of which mutually enhanced each other and led to apoptosis and autophagy in human OS cells in vitro and in vivo.

Activation of apoptosis-related signal transduction pathways is critical for cancer therapy. Caspase-4, a member of the caspase-1 subfamily, localizes to the ER membrane and is closely associated with ER stress-induced apoptosis, which enables the initiation of a protease cascade [14, 35]; its expression is significantly increased after CYT997 treatment. Western blot and immunohistochemical analyses confirmed that CYT997 increased levels of the apoptosis-related protein c-PARP. Using flow cytometry, we clearly showed that CYT997 induced apoptosis in a dose-dependent manner. Autophagy is a process involving the enclosure of cytoplasmic proteins or organelles into vesicles and subsequent fusion of the vesicles with lysosomes to form autolysosomes to degrade and recycle the contents of cells. In our study, we found an increase in the number of autophagosomes and the LC3B-II to LC3B-I ratio, indicating CYT997-induced autophagy in OS cells. However, in a study by Yong Teng [36], prostate cancer cells treated with CYT997 did not undergo autophagy, though this discrepancy may be related to cell line differences. Furthermore, autophagy has a dual role during treatment, namely, promoting cell survival or contributing to cell death [37]. In Gao’s study, CYT997 induced apoptosis and autophagy in human head and neck squamous cell carcinoma, and blockade of autophagy sensitized HNSCC to CYT997 [32]. The ATG family is an important protein in the process of autophagy [38]. And ATG5 is indispensable for autophagic vesicle formation, and ATG7 is the initiator of two autophagy pathways involving ATG12 and ATG8 [39, 40]. In our study we used shRNA to knockdown the ATG5 and ATG7. Discovered that autophagy activated by CYT997 tends to have a protective role, as inhibition of autophagy significantly enhanced the CYT997-induced apoptosis rate in vitro and in vivo. Taking these findings into consideration, we hypothesized that CYT997 may be a potent antitumor agent for OS. However, the underlying mechanism still needs to be explored.

The ER is an organelle that continuously undergoes highly dynamic rearrangements. There are three mechanisms for ER tubule motility. The first is the “membrane sliding mechanism”, whereby the ER slides along microtubules using a microtubule motor. The second is the “microtubule movement mechanism” in which the ER attaches to microtubules and follows the movement of the microtubules. The third is the “tip attachment complex (TAC) mechanism” that involves ER attachment to the plus end of the microtubule [41–44]. Microtubules are closely related to the ER, and the use of nocodazole to depolymerize microtubules leads to ER structural damage [45, 46]. Therefore, we hypothesized that microtubule-targeting agents can destroy the ER structure. In this study, we found a large amount of swollen, irregular ER in the cytoplasm when we employed TEM to observe autophagosomes. Using western blotting to verify UPR-related proteins, we discovered that CYT997 induced ER stress in OS cells in a time- and dose-dependent manner. The UPR pathway can activate apoptosis and autophagy via the CHOP-mediated Bcl-2 and IRE1α pathway [47, 48].To explore the relationship of CYT997-induced ER stress with apoptosis and autophagy, we used GSK2606414 to inhibit PERK and block the UPR pathway and found that both apoptosis and autophagy were reduced. Thus, we consider CYT997-induced apoptosis and autophagy to be downstream of ER stress.

Normally, ROS can function as signals to regulate cell proliferation and survival, whereas a disproportionate amount of ROS can damage cellular components, disturb normal cellular processes and lead to cell death [49, 50]. One of the remarkable features of CYT997-treated OS cells was the generation of ROS. To investigate whether ROS play a potential role in ER stress, we used NAC to decrease oxidative stress and found that ER stress was alleviated, suggesting that the ER stress induced by CYT997 is partially dependent on ROS. Under physiological conditions, ROS are often produced by the mitochondrial redox chain, but under pathological conditions, the ER also produces a large amount of ROS [51, 52]. ERO1 is an oxidative protein-folding enzyme that accepts electrons from reduced PDI and transfers them to molecular oxygen to generate H2O2, resulting in ROS production [52, 53]. Based on our results, both blockade of the UPR pathway and inhibition of ERO1 significantly reduced the level of ROS; overexpression of ERO1 markedly enhanced the level of ROS despite inhibition of the UPR pathway, indicating that the PERK/eif2α/CHOP/ERO1 axis plays an important role in CYT997-induced ROS production. As blockade of ER stress does not completely eliminate ROS, we speculated that CYT997 also damages mitochondria. Therefore, we measured the MMP with the fluorescent mitochondrial probe JC-1 and observed an obvious decrease in the red-green fluorescence ratio after CYT997 treatment, indicating that CYT997 damaged the mitochondria and produced ROS in OS cells. Elamipretide is a mitochondrial-targeted antioxidant peptide that could reduce the ROS produced by redox chain [54]. We found that autophagy and apoptosis were reduced after elamipretide treatment, the expression of proteins related to ER stress was also reduced. We suggest that the ROS in CYT997-treated cells are produced by both the ER and mitochondria. Using the above evidence, we demonstrated that the ER stress induced by CYT997 could increase ROS production and that ROS could aggravate ER stress. Therefore, we believe that the ER stress and ROS production caused by CYT997 treatment exacerbate each other and promote cell death.

## Conclusion

We identified the antitumor effects of CYT997 on OS in vitro and in vivo and the potential molecular mechanisms. We found that CYT997 significantly increased apoptosis and autophagy via ROS and the ER stress pathway and that CYT997-induced ROS and ER stress mutually reinforced each other. We also demonstrated that CYT997 significantly decreased tumor growth in vivo without obvious toxicity. Our study indicates that CYT997 may be a novel drug candidate for the treatment of OS.

## Additional files

Additional file 1: **Figure S2**. The histogram of the WB results. *P < 0.05, significantly different compared with the control group. # *P* < 0.05, significantly different compared with the CYT997 single treatment group. (TIF 26748 kb)

Additional file 2: **Figure S1**. (A) 143B cells transfected with ATG5shRNA were treated with different concentrations of CYT997 for 24 h, followed by cell proliferation detection using CCK-8 assays. (B) 143B cells were transfected with ATG5-targeted shRNA and then treated with or without 80 nM CYT997 for 24 h. Levels of apoptosis- and autophagy-related proteins were detected by western blotting. (C) 143B cells transfected with ATG7shRNA were treated with different concentrations of CYT997 for 24 h, followed by cell proliferation detection using CCK-8 assays. (D) 143B cells were transfected with ATG7-targeted shRNA and then treated with or without 80 nM CYT997 for 24 h. Levels of apoptosis- and autophagy-related proteins were detected by western blotting. (E)143B cells were co-treated with CQ (10 mM) and different concentrations of CYT997 (80 nM) for 24 h, followed by cell proliferation detection using CCK-8 assays. *P < 0.05, significantly different compared with the control group. # *P* < 0.05, significantly different compared with the CYT997 single treatment group. (TIF 23312 kb)

## Abbreviations

3-MA: 3-methyladenine
AVOs: acidic vesicular organelles
CQ: chloroquine
DCFH-DA: 2′,7′-dichlorodihydrofluorescein diacetate
DMSO: dimethyl sulfoxide
Ela: elamipretide
ER: endoplasmic reticulum
ERO1: endoplasmic reticulum oxidoreductin 1
FBS: fetal bovine serum
NAC: N-acetyl-L-cysteine
OS: osteosarcoma
PBS: phosphate-buffered saline
ROS: reactive oxygen species
UPR: unfolded protein response
