# Supplementary figures and images for: CYT997(Lexibulin) induces apoptosis and autophagy through the activation of mutually reinforced ER stress and ROS in osteosarcoma
Source: J Exp Clin Cancer Res. 2019 Jan 31;38:44. doi: 10.1186/s13046-019-1047-9 (PMC6357486; doi:10.1186/s13046-019-1047-9)

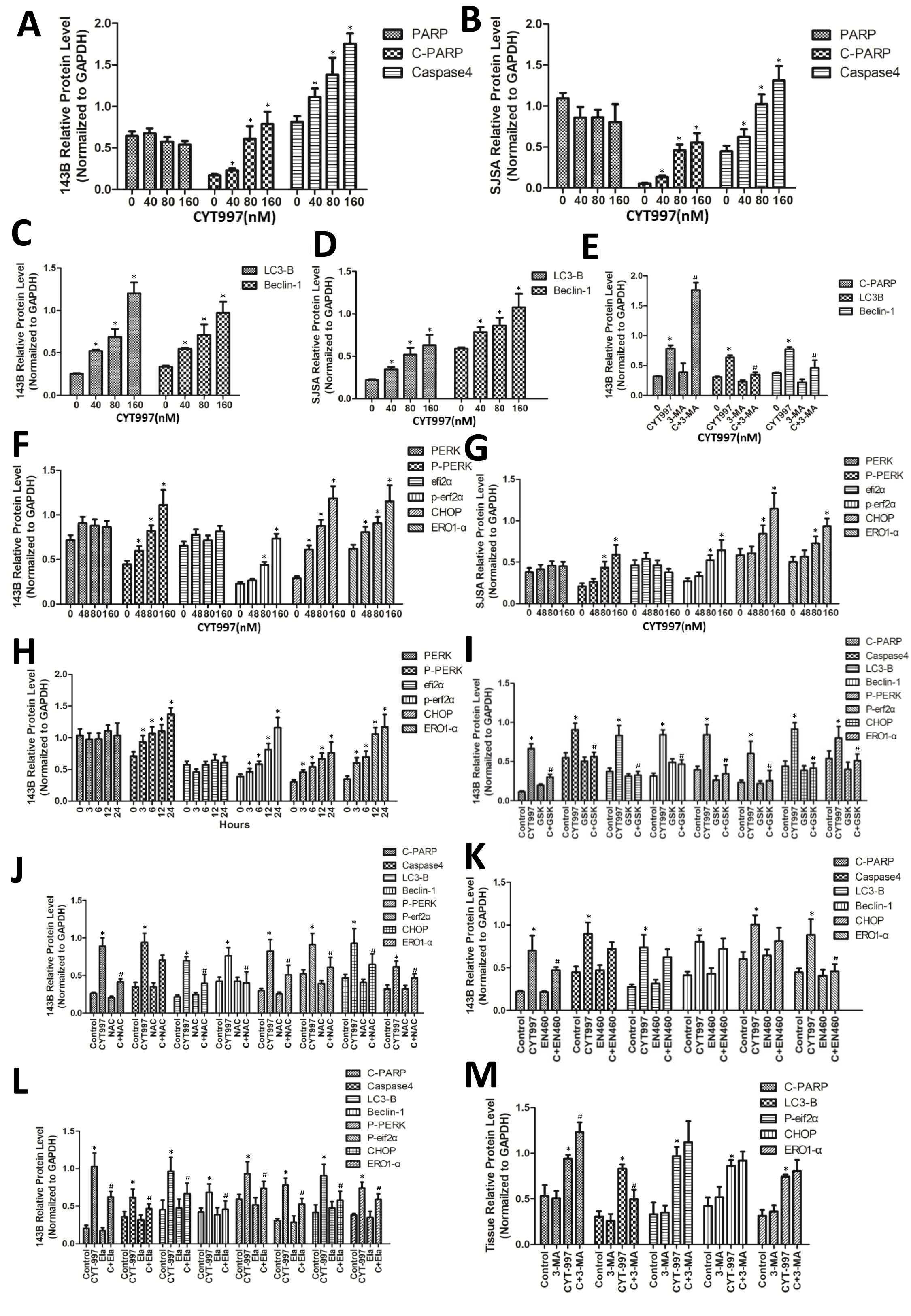

Supplement: Supplementary file 1 — Figure S2. The histogram of the WB results. *P < 0.05, significantly different compared with the control group. # P < 0.05, significantly different compared with the CYT997 single treatment group. (TIF 26748 kb) [file 13046_2019_1047_MOESM1_ESM.tif]

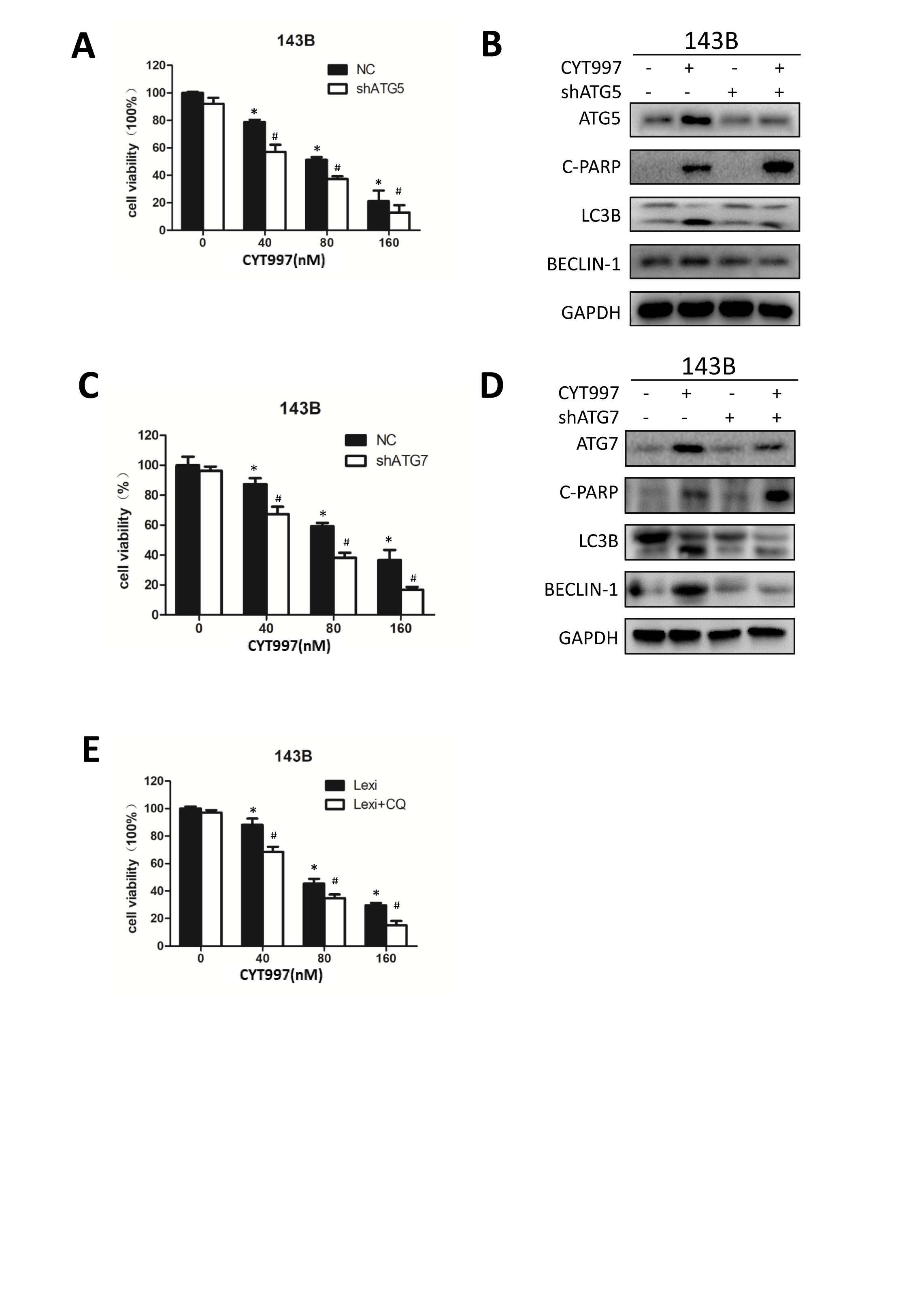

Supplement: Supplementary file 2 — Figure S1. (A) 143B cells transfected with ATG5shRNA were treated with different concentrations of CYT997 for 24 h, followed by cell proliferation detection using CCK-8 assays. (B) 143B cells were transfected with ATG5-targeted shRNA and then treated with or without 80 nM CYT997 for 24 h. Levels of apoptosis- and autophagy-related proteins were detected by western blotting. (C) 143B cells transfected with ATG7shRNA were treated with different concentrations of CYT997 for 24 h, followed by cell proliferation detection using CCK-8 assays. (D) 143B cells were transfected with ATG7-targeted shRNA and then treated with or without 80 nM CYT997 for 24 h. Levels of apoptosis- and autophagy-related proteins were detected by western blotting. (E)143B cells were co-treated with CQ (10 mM) and different concentrations of CYT997 (80 nM) for 24 h, followed by cell proliferation detection using CCK-8 assays. *P < 0.05, significantly different compared with the control group. # P < 0.05, significantly different compared with the CYT997 single treatment group. (TIF 23312 kb) [file 13046_2019_1047_MOESM2_ESM.tif]
